# Melatonin: Buffering the Immune System

**DOI:** 10.3390/ijms14048638

**Published:** 2013-04-22

**Authors:** Antonio Carrillo-Vico, Patricia J. Lardone, Nuria Álvarez-Sánchez, Ana Rodríguez-Rodríguez, Juan M. Guerrero

**Affiliations:** 1 Institute of Biomedicine of Seville (IBiS) and Department of Medical Biochemistry and Molecular Biology, Virgen del Rocío University Hospital/CSIC/University of Seville, 41013 Sevilla, Spain; E-Mails: vico@us.es (A.C.-V.); plardone@us.es (P.J.L.); 2 Institute of Biomedicine of Seville (IBiS) and Department of Clinical Biochemistry, Virgen del Rocío University Hospital/CSIC/University of Seville, 41013 Sevilla, Spain; E-Mails: nalvarez-ibis@us.es (N.Á.-S.); rodriguezana13m@gmail.com (A.R.-R.)

**Keywords:** melatonin, pineal, immune system, cytokines, inflammation, infection, autoimmunity, vaccination, immunosenescence

## Abstract

Melatonin modulates a wide range of physiological functions with pleiotropic effects on the immune system. Despite the large number of reports implicating melatonin as an immunomodulatory compound, it still remains unclear how melatonin regulates immunity. While some authors argue that melatonin is an immunostimulant, many studies have also described anti-inflammatory properties. The data reviewed in this paper support the idea of melatonin as an immune buffer, acting as a stimulant under basal or immunosuppressive conditions or as an anti-inflammatory compound in the presence of exacerbated immune responses, such as acute inflammation. The clinical relevance of the multiple functions of melatonin under different immune conditions, such as infection, autoimmunity, vaccination and immunosenescence, is also reviewed.

## 1. Introduction

As early as 1926, Berman reported improved resistance to infectious diseases in kittens fed for two years with pineal gland extracts from young bulls. Thus, the pineal-immunity relationship was already established prior to the discovery of melatonin in 1958 by Aaron Lerner *et al.*[1], who were attempting to isolate the pineal factor responsible for skin lightening in amphibians, which had been previously described by McCord and Allen in 1917. In recent decades, skepticism and perplexity regarding the relationship between melatonin and the pineal gland has been transformed by numerous and rigorous scientific analyses; now, this relationship has not only acquired scientific respectability, but is also of great physiological interest.

Melatonin (*N*-acetyl-5-methoxy-tryptamine) is an indoleamine that appeared very early during evolution. It is present in bacteria, unicellular eukaryotic organisms, invertebrates and vertebrates, algae, plants and fungi, and is also found in various edibles, such as vegetables, fruits, herbs and seeds [2]. Melatonin is converted from the amino acid tryptophan in two steps: first, from tryptophan into serotonin followed by acetylation via arylalkylamine *N*-acetyltransferase (EC 2.3.1.87; AA-NAT), and second, conversion to melatonin by hydroxyindole-*O*-methyl transferase (EC 2.1.1.4; HIOMT) [3]. The exclusive melatonin biosynthetic pathway reflects the high conservation of the molecule throughout many phylogenies. The production and release of melatonin from the pineal gland follows a circadian rhythm with a peak at night and the lowest levels during the light phase [4]. This temporal chemical signal is involved in the synchronization of several rhythmic physiological functions and is thus the subject of considerable clinical interest in terms of biological processes related to period and phase shifts, such as sleep disturbances, jetlag and shiftwork [5]. In addition to its chronobiotic function, melatonin exerts cyto-protective actions by regulating oxidative stress, apoptosis and mitochondrial homeostasis [6]. Furthermore, its oncostatic [7] and immunomodulatory activities [8], among many others, highlight the potential clinical relevance of melatonin.

The molecular mechanisms responsible for the pleiotropic effects of melatonin involve two main actions: binding to high-affinity G-protein-coupled receptors at the membrane level; and/or interaction with intracellular targets to modulate signal transduction pathways, redox-modulated processes, or the scavenging of free radicals [9].

Since the description of antibodies highly specific to melatonin in the mid-1970s [10], a substantial body of research has identified extrapineal sources of melatonin in a number of organs, tissues and cells, redefining the classic line of thought that melatonin is an exclusively pineal compound. Over the last 20 years, the gastrointestinal tract has been confirmed as an important source of melatonin [11], along with skin [12], retina and the Harderian gland [13]. Original studies from the last few decades have also identified *de novo* synthesis of melatonin by the immune system, which was reviewed in [14].

The immune system operates through a complex network of coordinated interactions involving numerous cells, proteins and molecules to protect the host against foreign agents that enter the body. The immune response is the result of two main types of immunity: the innate or non-specific response, and the acquired or specific response. The first includes defense mechanisms that are present even before infection occurs, facilitating a quick response. These mechanisms respond to microorganisms in the same way and with the same intensity, even with repeated infections. Innate immunity only involves the identification of specific structures shared by related groups of microorganisms, and it is unable to distinguish between subtle differences for substances that are recognized. The major cellular components of the innate response are macrophages, neutrophils, basophils, eosinophils and natural killer cells (NK), in addition to various soluble factors, such as the cytokines tumor necrosis factor-alpha (TNF-α), interleukin (IL)-1β, IL-6 and IL-8. In contrast to the innate response, the specific immune response is quite refined, and the magnitude of the response increases with successive exposures to a specific microorganism. T and B lymphocytes are the main components of the acquired immune response, in addition to circulating proteins such as antibodies and cytokines. Specific immunity consists of humoral and cellular immunity. The first is primarily mediated by antibodies, which recognize and bind to extracellular pathogens or non-self molecules, turning them into targets for destruction by macrophages, among other functions. Cellular immunity acts on intracellular microorganisms and is primarily mediated by cytotoxic T lymphocytes (CD8+), which recognize and destroy infected cells, and T helper lymphocytes (Th; CD4+), which are key elements in the regulation and coordination of the innate, humoral and cellular responses through the production of a large variety of cytokines. Based on the cytokine milieu, the expression of specific transcription factors and patterns of protein secretion, Th cells can differentiate primarily into four major phenotypes: Th1, Th2, Th17 (effector phenotype), and regulatory T (Treg) cells, which control excessive responses of the effector lineages. Th1 cells play a key role in the development of inflammatory processes through the production of cytokines such as IFN-γ. Th2 cells produce cytokines such as IL-4, IL-5, IL-10 and IL-13 and contribute to the regulation of the humoral and anti-inflammatory responses. Th17 cells, a novel subset of CD4+ T cells, have mainly been identified on the basis of RORγt transcription factor expression and the production of IL-17 [15]. In addition to their involvement in autoimmunity, Th17 cells eliminate extracellular pathogens, and their relevance in inflammatory processes is becoming increasingly apparent. Currently, Th1/Th17 responses are considered pro-inflammatory, while the Th2 response is considered anti-inflammatory. The description of Treg cells and their remarkable functions in the control of effector cells has also updated the field of immunology. These cells represent a unique subpopulation of CD4+ cells (mostly CD25+) for which the expression of the transcription factor Foxp3 is a hallmark [16].

## 2. Pineal-Immune System Cross-Talk: From the Pineal Gland to the Immune System and Return

A large body of evidence has shown a clear relationship between the neuroendocrine and immune systems. This link is illustrated by a bidirectional communication circuit in which the endogenous substances of the neuroendocrine system act on the immune system and *vice versa*. This network also shares a common language via compounds synthesized in both systems, such as acetylcholine, adrenocorticotropic hormone (ACTH), endorphin, vasoactive intestinal peptide (VIP), somatostatin and growth hormones [17].

The pineal gland and its main product, melatonin, are considered members of this network. A functional connection between this gland and the immune system has been widely described through two main experimental approaches: pinealectomy and rhythmic synchronization between melatonin synthesis and the immune system. Pineal ablation promotes massive weight loss in primary and secondary lymphoid organs and a decrease in the cellular components and functions associated with the innate and specific responses. Furthermore, synchronization between the rhythmicity of melatonin production and circadian and seasonal adjustments in the immune system has been widely reported; this is extensively reviewed in [14,18].

The pineal gland is also an immune target. Interferon-gamma (IFN-γ) was shown to increase the production of melatonin from *in vitro*-cultured rat pineal glands [19]. Administration of recombinant IL-1β inhibited serum melatonin levels in rats through a receptor-mediated mechanism [20], whereas granulocyte colony-stimulating factor (G-CSF) and granulocyte-macrophage colony-stimulating factor (GM-CSF) stimulated the synthesis of melatonin both *in vivo* and *in vitro*[21]. A study of surgically bursectomized chickens revealed the pivotal role of the bursa of Fabricius in the ontogeny of circadian melatonin synthesis, as a significant reduction in plasma melatonin levels and NAT activity was recorded after bursa of Fabricius ablation [22]. Immunization with sheep red blood cells (SRBCs) also caused changes in night NAT activity in chickens [23], whereas peritonitis promoted a downregulation of *Aa-nat* transcription and a subsequent decrease in nocturnal melatonin levels [24]. Recently, a series of elegant papers from the Markus group has outlined the hypothesis that mounting inflammatory responses involves the suppression of nocturnal melatonin production, reinforcing the idea of bidirectional pineal-immune system cross-talk. The transcription of *Aa-nat* together with the synthesis of the melatonin precursor *N*-acetylserotonin was transiently inhibited by administration of TNF-α to *in vitro*-cultured rat pineal glands [25]. Moreover, suppression of increased nocturnal melatonin in mothers with mastitis was highly correlated with increased TNF-α production [26]. Likewise, an increase in TNF-α levels after Caesarean section resulted in the suppression of serum melatonin nocturnal levels [27]. Additionally, lipopolysaccharide (LPS) treatment not only reduced the production of nocturnal melatonin in rats but also enhanced endothelial cell adherence, which was normalized after melatonin administration [28]. LPS was shown to induce TNF-α production in the rat pineal gland through activating toll-like receptor 4 (TLR-4) [29]. Subsequently, the production of TNF-α by pineal gland microglia was found to act on tumor necrosis factor receptor 1 (TNFR1), driving the nuclear translocation of NF-κB, which represses *Aa-nat* transcription and in turn suppresses melatonin synthesis [30].

A main feature of the neuroendocrine–immune network is the use of a common language. To this end, the immune system endogenously produces several peptidic and non-peptidic compounds, such as acetylcholine, adrenaline, neurotransmitters and neuroendocrine hormones, which are also typical of the neuroendocrine system [31]. A number of studies have suggested possible endogenous melatonin synthesis by the immune system. These studies have revealed much evidence for the notion that melatonin should not be solely described as a hormone according to the classic definition; this was reviewed in [32]. One such piece of evidence that melatonin can be distinguished from a classical hormone is that the direct presence of melatonin or key enzymes involved in its synthesis have been identified in different non-endocrine organs, such as retina, lens, Harderian gland, gut cells [11,33], skin [12] and many others [34,35].

Thus, evidence is emerging that the immune system is one source of extrapineal melatonin. At the end of the 1980s, the works of Finocchiaro suggested that human peripheral blood mononuclear cells (PBMCs) possessed the capacity to produce melatonin *in vitro* after being cultured in the presence of IFN-γ or serotonin [36,37]. In early 2000, two studies revealed the presence of high concentrations of melatonin in rat, mouse and human bone marrow [38,39]. Tan *et al.* described the presence of NAT and HIOMT activities in bone marrow cells and noted that significantly higher concentrations of melatonin were still present in rat bone marrow after pinealectomy, indicating *de novo* synthesis. Similar results were described by Conti *et al.* with human bone marrow cells and in different mouse strains. High concentrations of melatonin in long-term bone marrow cultures (four weeks) also suggested the endogenous synthesis of melatonin. Rat peritoneal macrophages also produce melatonin *in vitro* after incubation with tryptophan [40]. In addition to the aforementioned sources, melatonin and/or its biosynthetic machinery have been located in a variety of immune tissues, organs and cells, such as rat, mouse and human thymus [41,42]; spleen, bone marrow and circulating leukocytes [43]; mast cells, natural killer cells and eosinophils [34]; and in several immune cell lines [36,39,44,45].

An important fact that also supports the relationship between melatonin and the immune system is the presence of melatonin receptors in a wide variety of organs and immune cells from various species of mammals and birds. Currently, there is enough evidence to affirm that melatonin not only interacts with membrane-associated and intracellular targets, but also that this interaction mediates important regulatory effects on the immune system (Table 1).

Despite the relatively high numbers of putative melatonin sources within the immune system, knowledge of the physiological actions of local immune-derived melatonin is limited. We were the first to report that *in vitro-*cultured human lymphocytes not only actively synthesize and release substantial amounts of melatonin [73], but that this melatonin modulates the IL-2/IL-2 receptor (IL-2R) system via receptor-mediated intra-, auto- and/or paracrine actions [51]. The use of luzindole, a melatonin membrane receptor antagonist, potentiated the inhibitory effects of PGE_2_ on IL-2 production by Jurkat cells, demonstrating the membrane receptor-mediated effects of endogenous melatonin [44]. To further investigate the role of immune-derived melatonin in the IL-2/IL-2R system, HIOMT and MT_1_ knock-down Jurkat cells were used. Both low levels of HIOMT activity and MT_1_ expression diminished IL-2 production after phytohaemagglutinin (PHA) activation [52]. Colostrum immunocompetent cells produced a transient surge of melatonin after *in vitro* stimulation by enteropathogenic *Escherichia coli* or zymosan, suggesting the involvement of local melatonin in modulating the phagocytic capacity of the cells [26]. This is in accordance with the high concentrations of melatonin we recorded in PHA-stimulated PBMCs [73]. Additionally, we confirmed that high levels of melatonin after optimal PHA stimulation impaired exogenous melatonin action in the production of IL-2, likely via a binding site saturation mechanism [51]. The existence of individuals with different immune cell sensitivities to melatonin and the impaired melatonin action against an LPS-stimulated inflammatory response observed in human monocytes during winter darkness (70 degrees N) [74] also support the notion of a masking effect for endogenously synthesized melatonin exerted by the immune system. Similarly, the presence of high amounts of endogenous melatonin may be the reason that many authors are able to demonstrate *in vitro* effects for exogenous melatonin only when cells either are not or are only slightly activated [71,75]. Opposing functions for NF-κB in the transcriptional control of *Aa-nat* expression have recently been described in pinealocytes *versus* macrophages, which may represent an interesting switch mechanism in the regulation of pineal *versus* immune-derived melatonin production under inflammatory conditions [76].

## 3. Pleiotropic Actions of Melatonin Administration on the Immune Response

A large amount of evidence has demonstrated the immunomodulatory capacity of melatonin administration in both *in vivo* and *in vitro* models [14]. Some studies have shown that melatonin treatment promotes an increase in the weight of immune organs, both under basal and immunosuppressed conditions (Table 2) [77–80]. Conversely, the anti-proliferative effects of melatonin have been observed *in vitro* in PHA-stimulated human lymphocytes [81]. Melatonin also modulates both the innate and specific immune responses through regulation of immunocompetent cell proliferation [82,83] and secretion of immune mediators, such as cytokines [18].

### 3.1. Immunomodulatory Actions of Melatonin in the Innate Immune Response

Melatonin modulates the main cellular components of the innate immune response. Multiple daily melatonin injections into the pineal glands and surrounding areas in rat brains have been shown to promote a significant increase in macrophage/microglia cellularity [84]. NK cells and monocytes were also increased in the bone marrow of healthy young mice fed with melatonin [85]. Similarly, the administration of melatonin to healthy subjects promoted stimulation of NK cell activity [86], which was also enhanced in the spleens and axillary nodes of 24-month-old female rats treated with melatonin [87]. Additionally, it has been demonstrated that the chemotaxis index is significantly boosted by melatonin. The chemotactic effects that melatonin exerts on leukocytes were revealed with healthy human neutrophils and PBMCs in response to *in vitro* melatonin application [88]. Healthy mice receiving 10 consecutive daily intraperitoneal injections showed enhanced antigen presentation by splenic macrophages to T cells, which simultaneously occurred with increased MHC class II expression and production of IL-1 and TNF-α [89]. Melatonin is also able to enhance the phagocytic capacity of heterophils [90].

An early hallmark of the inflammatory response is the cascade production of TNF-α, IL-1β and IL-6 by activated macrophages. These cytokines are considered to be serum biomarkers of inflammation. Splenocytes from healthy mice treated with a very high concentration of melatonin (500 mg/kg) produced increased levels of IL-1β, even without *in vitro* mitogenic stimulation [91]. Experimental trauma-hemorrhage in mice gave rise to an immunosuppressed state with low levels of IL-1 and IL-6 production, which were restored to basal control levels after melatonin treatment [92]. The *in vitro* incubation of human monocytes in the presence of melatonin promoted a pre-activation state, improving the cytotoxic response to LPS [93]. Melatonin also enhanced IL-6 and IL-12 production by cultured monocytes, but only under suboptimal stimulation [71,75]. Conversely, melatonin demonstrates alternate behavior in situations with exacerbated immune responses. Melatonin reduced neutrophil infiltration and levels of inflammatory mediators during rat heatstroke-induced lung inflammation and airway hyperreactivity [94,95]. Decreased neutrophil infiltration was also one of the mechanisms by which melatonin protected against experimental acute pancreatitis [96]. Melatonin administration upon reperfusion decreased the migration of circulatory neutrophils and macrophages/monocytes into injured brain and inhibited microglial activation following transient focal cerebral ischemia in rats [97]. In addition to chemotaxis, endothelial adherence and permeability is a key process involved in the recruitment of immune cells towards targets. Injection of melatonin was found to reduce leukotriene B4-induced endothelial cell hyper-adhesiveness, suggesting a reduction in the capacity of vascular cells to interact with neutrophils [98]. The endothelial cell barrier-protective effects of melatonin have also been described in human umbilical vein endothelial cells with increased permeability induced by IL-1β [99]. Melatonin suppressed LPS-induced cyclooxygenase-2 (COX-2) and inducible nitric oxide synthase (iNOS) protein levels in the murine macrophage cell line Raw264.7 [100]. A microarray analysis of gene expression in LPS-activated Raw cells confirmed the melatonin-induced anti-inflammatory profile [101]. Melatonin also downregulated chemokine expression in the BV2 murine microglial cell line following LPS stimulation [102]. Melatonin and its derivative AFMX suppressed the production of IL-8, a neutrophil chemotactic factor, in human pulmonary fibroblasts [103] and LPS-activated peripheral blood neutrophils [104]. A biphasic effect of melatonin on the phorbol myristate acid (PMA)-induced respiratory burst in human neutrophils has also been described; while low doses (10 nM) increased the response, high doses (2 mM) inhibited it [105]. Additionally, melatonin neutralized the exacerbated production of pro-inflammatory mediators, mainly cytokines, in a large number of *in vivo* models of inflammation (Table 3).

*In vitro* approaches have also highlighted the molecular mechanisms involved in the anti-inflammatory actions of melatonin. Melatonin inhibited LPS-stimulated TNF-α, IL-1β, IL-6, IL-8 and IL-10 production in Raw264.7 cells through a mechanism involving the attenuation of NF-κB activation [143]. Similarly, melatonin suppressed the production of LPS-activated IL-6 and NO in *Prevotella intermedia* by blocking NF-κB signaling through the inhibition of the nuclear translocation and DNA-binding activities of the NF-κB p50 subunit and suppression of STAT-1 signaling [144]. Additionally, melatonin decreased TLR3-mediated TNF-α and iNOS expression via inhibition of NF-κB activation in respiratory syncytial virus-infected Raw264.7 cells [145]. Melatonin also attenuated both the methamphetamine-induced upregulation of TNF-α, IL-1β and IL-6 in the HAPI rat microglial cell line [146] and the amyloid-beta-induced over-production of TNF-α and IL-6 in organotypic hippocampal cultures [147]. In conclusion, numerous studies have demonstrated that melatonin is an anti-TNF-α compound, which should be taken into account for future clinical trials. Similarly, a protective effect for melatonin in TNF-α-induced muscle atrophy in L6 myotubes was recently described [148].

### 3.2. Immunomodulatory Actions of Melatonin in the Specific Immune Response

Broadly speaking, melatonin exerts positive effects on the cellular and humoral responses under basal or immunosuppressed conditions. As early as 1986, Maestroni *et al.* reported that reconstitution of the nighttime plasma melatonin peak completely abrogated the humoral and cellular responses in propranolol-immunosuppressed mice [109]. Mice immunosuppressed by lead recovered splenic CD4+ cell numbers and functions after melatonin treatment [110]. Melatonin also averted age-induced immunosuppression in rats by increasing IgG1 and IgM levels [106]. Furthermore, melatonin significantly restored both dexamethasone- and aging-induced immunosuppression in squirrels [59,80,107]. Melatonin also increased B cell proliferation and the Th1 response (IL-2 and IFN-γ production) and decreased Th2 cytokines such as IL-10 in old mice [108].

Early *in vitro* studies suggested that melatonin has pro-Th1 effects [75]. Sub-stimulated PBMCs displayed enhanced production of Th1 cytokines, such as IFN-γ and IL-2, after *in vitro* melatonin treatment [71,75]. The diurnal rhythmicity of human cytokine production indicated that the IFN-γ/IL-10 peak occurs during the early morning; this peak positively correlated with plasma melatonin [149], suggesting a melatonin/Th1 causality. Splenocyte proliferation in response to the T cell mitogen concanavalin A was also enhanced by the addition of melatonin *in vitro*[150].

Conversely, melatonin significantly reduced the splenic CD19+ B-cell population in mice with experimental membranous nephropathy and diminished the overexpression of TNF-α, IL-1β and IFN-γ [151]. Further *in vivo* studies have shown the capacity of melatonin to promote a Th2 response in several models. The first report demonstrated that high doses of melatonin enhanced the production of the hallmark Th2 cytokine IL-4 in bone marrow lymphocytes [152]. Early nocturnal sleep induced a shift in the Th1/Th2 cytokine balance towards increased Th1 activity, whereas the Th2 response dominated during late sleep. A robust decrease in TNF-α-producing CD8+ cells was also observed during sleep [153], suggesting a correlation between melatonin and the Th2 response. Likewise, the absence of melatonin due to pinealectomy polarized rat thymic Th1/Th2 cells towards a Th1 response by increasing the production of IFN-γ and reducing IL-10 levels, implying that melatonin skews the immune response towards Th2 dominance [154]. Chronic administration of melatonin to antigen-primed mice increased the production of IL-10 and decreased the secretion of TNF-α, suggesting a Th2 response [155]. Melatonin inhibited the Th1 response by suppressing IFN-γ and IL-12 in mice with contact hypersensitivity [156]. Furthermore, melatonin protected against experimental reflux esophagitis by suppressing the Th1-mediated immune response [58]. Melatonin also acted as an immunosuppressive agent and reduced Th1 cytokine levels in an experimental model of ovarian transplant in mice, permitting prolonged graft survival [157].

The modulation of cytokines by melatonin in several *in vivo* models also supports an anti-Th1 response. Melatonin increased IL-10 production both locally and systemically in an experimental mouse model of septic shock [158]. Melatonin also significantly reversed both aging- and ovariectomy-induced reductions in IL-10 levels in rats [122]. Moreover, melatonin suppressed the development of experimental atopic dermatitis in NC/Nga mice by reducing total IgE, IL-4 and IFN-γ levels released from activated CD4+ cells [159]. It was found that the stimulation of IL-10 production is one mechanism by which melatonin exerts beneficial effects in acute pancreatitis [96]. Suppressed melatonin synthesis due to light exposure in mice increased IgM, IgG2a, IgG2b and IgG3 antibody levels after immunization with T cell-dependent antigens, while IgG1 antibody levels were significantly decreased [160]. Because IgG2a and IgG1 are typical hallmarks of the Th1 and Th2 responses, respectively, these results might support the idea of a Th1-biased effect with a melatonin deficit. Although there are no reports that have explored Th17 cells in relation to melatonin, we recently identified the Th17 lineage-specific transcription factor RORα as a natural target of melatonin in T cells [69]. RORα, along with RORγ, regulates Th17 cell differentiation [161], whereas the downregulation of RORα expression has been shown to be part of the typical Treg transcriptional signature [162]. A preliminary study highlighted the *in vivo* inhibitory actions of melatonin on Treg cell generation in cancer patients [163]. Additionally, the *in vivo* administration of melatonin to mice subjected to experimental cancer via inoculation of a foregastric carcinoma cell line was associated with the downregulation of CD4+CD25+ Treg cells and Foxp3 expression in the tumor tissue [164].

## 4. Clinical Relevance of Melatonin

### 4.1. Melatonin and Infection

**Scheme 1:**
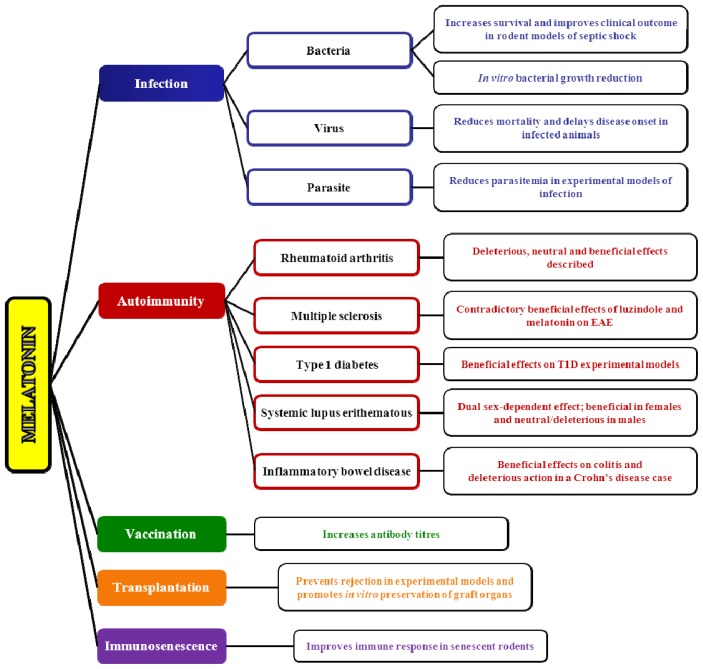
Melatonin in immune intervention.

In recent decades, melatonin has been reported to possess important functions as an antiviral, antibiotic and anti-parasitic molecule [165,166] (Scheme 1). In addition, a substantial body of research has highlighted the increased survival of animals subjected to septic shock after the administration of melatonin. The beneficial effects of melatonin manifest through its pleiotropic properties as an immunomodulator [18], antioxidant [167] and cyto-protector [158].

#### 4.1.1. The Role of Melatonin in Viral Infections

Increasing doses of melatonin (0.25 to 1 mg/kg) administered to mice infected with Venezuelan equine encephalomyelitis virus (VEEV) were found to reduce mortality up to 16% compared to 100% mortality in non-melatonin-treated mice [168]. Melatonin reduced viral levels in the brains of immunocompetent mice but not in immunosuppressed mice, suggesting that melatonin requires the integrity of the immune system for antiviral activity [169]. Additionally, melatonin significantly increased serum levels of TNF-α, IL-1β and IFN-γ. Blockage of IL-1β with anti-murine IL-1β antibodies completely neutralized the protective role of melatonin, suggesting that IL-1β is the main target for the rapid viral clearance induced by melatonin [170]. Melatonin also increased IL-1β levels in the brains of infected animals; in contrast, indoleamine reduced the production of TNF-α, contributing to the localized control of the inflammatory response in the brain [171]. In addition to modulating cytokine production, melatonin treatment diminished high nitrite concentrations and reduced the amounts of lipid peroxidation products in the brains and sera of infected mice [172].

Administration of melatonin prevented paralysis and death in mice infected with sub-lethal doses of encephalomyocarditis virus (EMCV) after acute stress [173]. Melatonin also reduced mortality due to persistent parvoviral infection (Aleutian disease) in mink, which results in marked hypergammaglobulinemia and immune complex-mediated tissular lesions [174]. The antiviral activity of melatonin was also evaluated in normal mice inoculated with Semliki Forest virus (SFV) and in stressed mice injected with attenuated non-invasive West Nile virus (WN-25). Administration of daily melatonin starting three days before virus inoculation and continuing 10 days after inoculation reduced viremia, significantly reduced mortality, and postponed the onset of disease and death [175].

The experimental murine acquired immune-deficiency syndrome (AIDS) induced by LP-BM5 leukemia retrovirus induces inhibition of Th1 cytokine release, stimulates Th2 cytokine secretion, enhances hepatic lipid peroxidation and causes vitamin E deficiency. Administration of dehydroepiandrosterone (DHEA) and/or melatonin prevented the reduction of B and T cell proliferation and Th1 cytokine secretion in female C57BL/6 mice with AIDS [176]. In terms of AIDS caused by human immunodeficiency virus type I (HIV-1), a positive correlation between serum levels of melatonin and IL-12 has been demonstrated. Additionally, a negative correlation between melatonin and plasma HIV-1 RNA levels has been observed; supporting this negative correlation, levels of serum melatonin in HIV-1-infected individuals were found to be significantly lower than in healthy controls [177].

#### 4.1.2. The Role of Melatonin in Bacterial Infections

The protective actions of melatonin against bacterial infections have been evaluated for the cultured H37Rv strain of *Mycobacterium tuberculosis* (*M. tuberculosis*). Melatonin supplied at a concentration of 0.01 mM, together with isoniazid, a frontline drug used in the treatment of tuberculosis, inhibited bacterial growth three to fourfold more than either of these compounds alone [178]. When intracellular bacterial growth was examined in infected monocytes, the addition of either isoniazid or melatonin alone did not have any effect on macrophage mortality or viability. However, their combination resulted in a marked reduction in bacterial loading. The microbicidal action of melatonin might be attributed to its capacity to form stable radicals that could either modify isoniazid action or bind to the mycobacterial cell wall, resulting in destabilization of the cell wall and causing enhanced permeability to isoniazid molecules. Mean plasma melatonin and aMT6 levels are lower in patients infected by *M. tuberculosis* than in control subjects [179]. Some studies have also shown that peaks in *M. tuberculosis* infection coincide with both the end of winter and the beginning of the summer season [180,181], suggesting that seasonal changes in the immune system caused by annual fluctuations in melatonin levels could be involved [179,182]. The microbicidal activity of melatonin has also been evaluated in chlamydial infections. The major defense mechanism against chlamydial infection is the synthesis of IFN-γ by the host and the subsequent induction of indoleamine 2,3-dioxygenase, resulting in the depletion of tryptophan from human cells [183]. Therefore, a deficit in melatonin production promoted by tryptophan depletion would be expected in chlamydial infections. *In vitro* administration of melatonin to three Chlamydiaceae species, *Chlamydia trachomatis*, *Chlamydophila pneumoniae* and *Chlamydophila felis*, reduced infection by 50%. This reduction was neutralized by pertussis toxin, an inhibitor of G proteins [184], which suggests a membrane receptor-mediated mechanism. An additional study demonstrated the *in vitro* anti-microbial activity of melatonin against multidrug-resistant Gram-positive and Gram-negative bacteria, methicillin-resistant *Staphylococcus aureus*, carbapenem-resistant *Pseudomonas aeruginosa* and *Acinetobacter baumannii*[185]. These results have clinical importance, as these strains have recently emerged as primary nosocomial pathogens in hospital outbreaks.

Sepsis encompasses a heterogeneous group of syndromes characterized by impaired body temperature, hypotension, vascular and myocardial dysfunction, hypoperfusion associated with cellular damage and, in severe cases, multiple organ failure [186]. It is a systemic response to the release of bacterial endotoxins, mainly LPS, that activates polymorphonuclear cells (PMN), monocytes and lymphocytes. The release of cytokines, especially TNF-α, IL-1β, IL-6, IL-12 and IFN-γ, and the generation of free radicals by PMNs and infiltrated macrophages in the tissues can lead to microvascular dysfunction and organ failure [186]; TNF-α and NO are essential mediators in the pathogenesis of sepsis and associated complications [187].

Several studies using experimental models of endotoxin-induced sepsis and multibacterial sepsis have demonstrated the important protective role of exogenous melatonin [158,187–192]. Supporting these studies, exposure to short photoperiods with the subsequent increase in endogenous melatonin was found to augment the survival of septic Siberian hamsters and to diminish TNF-α levels compared to animals exposed to long photoperiods [193].

The mechanisms of melatonin action in sepsis reflect the pleiotropic capacity of the molecule. Melatonin blocks the overproduction of pro-inflammatory cytokines, especially TNF-α [158,190,194–196], and increases IL-10 levels [158,192,196]. In addition, melatonin was found to increase the weight of the spleen in endotoxin-septic rats compared with control animals [191] and to counteract sepsis-induced apoptosis in the spleen [158]. Moreover, melatonin neutralised inflammatory infiltration in different tissues of septic animals [197,198].

The beneficial effects of melatonin are not confined to direct modulation of the immune system. Indeed, melatonin increases total antioxidant capacity [194] and/or reduces the production of reactive oxygen species (ROS) and reactive nitrogen species (RNS) and their deleterious effects on biomolecules in septic rodents [194,199,200]. Melatonin also protects against sepsis-induced damage of the mitochondria by restoring impaired mitochondrial antioxidant systems, enhancing both glutathione (GSH) levels and glutathione reductase (GRd) activity, inhibiting nitrite formation and inducing mitochondrial iNOS expression. Additionally, melatonin reduces mitochondrial lipid peroxidation in different tissues and decreases sepsis-induced alterations in mitochondrial respiratory chain activity and ATP synthesis [187,201].

As with most inflammatory conditions that have been tested, the intracellular actions of melatonin in experimental models of sepsis involve the reduction of NF-κB nuclear translocation [196,202]. Melatonin also reduces p38 MAPK and poly ADP ribose synthase (PARS) activation, which is activated by DNA damage caused by ROS and RNS [202,203]. The effects of melatonin can be, at least in part, mediated by binding to the membrane receptors MT_1_ or MT_2_, as luzindole blocks melatonin-induced decreases in pro-inflammatory cytokines [190].

In humans, septic patients hospitalized in intensive care units (ICUs) were found to have altered circadian rhythms for 6-sulfatoxymelatonin (aMT6) urine excretion, with loss of circadian periodicity, diminished phase amplitude and delayed acrophase [204], but light exposure in the ICU was not responsible for this impairment in urine aMT6 excretion [205]. Additionally, nocturnal plasmatic melatonin levels were found to inversely correlate with illness severity in ICU patients with severe sepsis [206]. In a similar study performed in pediatric patients, nocturnal melatonin concentrations in children with sepsis in a septic shock state were significantly higher than those of septic patients not in a septic shock state. However, there was no significant difference in nocturnal aMT6 excretion between septic patients, with or without septic shock, and non-septic patients. Interestingly, 24-h aMT6 excretion in septic patients with liver dysfunction was significantly lower than in septic patients without liver dysfunction. Additionally, nocturnal melatonin concentrations in non-survivors were significantly higher than in survivors, whereas total aMT6 excretion in non-survivors was significantly lower than in survivors [207,208]. The parallel measurement of serum melatonin together with the urine levels of aMT6 would provide convenient data to allow disturbances in aMT6 due to hepatic dysfunction to be discarded. Exogenous melatonin has also been shown to improve clinical outcome for septic newborns by reducing lipid peroxidation, white cell counts, neutrophil counts and C-reactive protein levels [209]. Moreover, melatonin reduces pro-inflammatory cytokine production (IL-6, IL-8, TNF-α), lipid peroxidation and nitrite and nitrate levels in newborns suffering from respiratory distress syndrome [142].

#### 4.1.3. The Role of Melatonin in Parasite Infections

Some studies have shown that TNF-α, IFN-γ and IL-12 are important for the control of *Trypanosoma cruzi* (*T. cruzi*) infection by ensuring the induction of an efficient adaptive host response [210]. Therefore, several reports have examined the effects of melatonin combined with other drugs in the control of experimental models of *T. cruzi* infection. Combined treatment with melatonin and meloxicam was found to reduce the levels of parasitemia and increase TNF-α, IFN-γ and IL-2 production [211]. In conjunction with DHEA, melatonin diminished the parasite load in blood and tissue [212]. Despite a strong and vigorous anti-parasite immune response, melatonin was not able to completely clear infection, enabling *T. cruzi* to persist in a majority of hosts [213]. After *T. cruzi* infection, the thymus, a central lymphoid organ able to generate mature T cells, undergoes a dramatic loss in size, resulting in a reduction in the number of thymocytes. A recent study found that zinc combined with melatonin therapy triggered thymocyte proliferation in rats inoculated with *T. cruzi* compared to untreated animals [214].

Many studies have demonstrated that Plasmodium falciparum (*P. falciparum*), a parasite that colonizes hepatocytes and red blood cells (RBCs) and causes the deadly disease malaria, depends on intracellular calcium. Authors have stated that melatonin and its precursors derived from the tryptophan catabolism induce calcium release and modulate the *P. falciparum* cell cycle [215].

### 4.2. Melatonin and Autoimmunity: A Winding Road

Some studies have implicated both endogenous and exogenous melatonin in the development of different autoimmune diseases (Scheme 1), including rheumatoid arthritis (RA), multiple sclerosis (MS), systemic lupus erythematosus (SLE), type 1 diabetes (T1D) and inflammatory bowel disease (IBD). However, results describing the relationships between melatonin and other autoimmune diseases are scarce. One study found a loss of nocturnal melatonin elevation and increased morning plasmatic melatonin levels in psoriasis patients [216]. Furthermore, melatonin reduced type II collagen-induced proliferation of lymphocytes in patients with autoimmune hearing loss [217] and protected against autoimmune glomerulonephritis in an experimental model of idiopathic membranous nephropathy [151].

#### 4.2.1. Melatonin and Rheumatoid Arthritis

The effects of melatonin on RA, a common autoimmune disease suffered by approximately 1% of the world’s population [218], is controversial. Different studies using experimental models of arthritis have suggested deleterious actions for both endogenous and exogenous melatonin. Animals maintained in constant darkness undergo more severe collagen-induced arthritis and exhibit higher titers of serum anti-collagen antibodies than those kept under constant light or a normal photoperiod [219]. The effects of constant darkness were reversed by pinealectomy [220]. Furthermore, administration of melatonin to mice immunized with rat collagen II and kept under constant light promoted more severe arthritis when it was injected at the beginning of the immunization, whereas melatonin injection at the onset of the disease (day 30–39) did not affect the clinical signs of the disease [221]. An additional study showed that melatonin increased serum anti-collagen antibody titers and IL-1β and IL-6 levels in the serum and joints of arthritic rats, while it decreased oxidative markers in serum but not in joints. As expected, pinealectomy had an opposite effect compared to melatonin, resulting in reduced antibodies, cytokine levels and oxidative stress in joints but also elevated oxidative markers in serum [222]. In contrast with those studies supporting the deleterious effects of melatonin, prophylactic and/or therapeutic treatment with melatonin reduced hind paw swelling similar to indomethacin in an adjuvant-induced arthritis model [223].

An increase in the incidence and severity of RA has been shown to be associated with higher latitudes, suggesting that augmented melatonin production during long winter nights could be related to RA. Supporting this notion, the nocturnal levels of melatonin in RA patients from Estonia were found to be higher than those in Italian patients [224]. Moreover, the risk of arthritis is inversely associated with UV-B exposition [225], which is radiation known to reduce pineal synthesis of melatonin [226]. RA symptoms are also worse in the early morning [224], coinciding with high levels of pro-inflammatory cytokines and low serum concentrations of cortisol [227]. Interestingly, some authors have reported a rise in serum melatonin levels during the early morning in RA patients compared with healthy controls, a positive correlation between melatonin levels and disease activity scores and an advance in the nocturnal melatonin peak compared to control subjects [228–230]. However, other authors have reported significantly lower levels of morning plasmatic melatonin [231] and increased nocturnal pineal production of melatonin induced by Freund’s adjuvant in an experimental model of arthritis [232].

*In vitro*, cultured RA synovial macrophages have high-affinity binding sites for melatonin [233] and produce high levels of IL-12 and NO after melatonin administration [234]. Additionally, synovial fluid from RA patients has relatively high levels of melatonin [233]. Conversely, melatonin inhibits the excessive proliferation of RA fibroblast-like synoviocytes through activation of the cyclin-dependent kinase inhibitors P21 (CIP1) and P27 (KIP1) mediated by ERK [235]. Fibroblasts from synovial membranes collected from RA patients also show impaired circadian expression of timekeeping genes and pro-inflammatory cytokines such as TNF-α, IL-1β and IL-6 [236].

Interestingly, the only study conducted in RA patients with active disease receiving daily melatonin at night over six months reported low antioxidant profiles in patients, increased neopterin concentrations and erythrocyte sedimentation rates (inflammation indicators) and no changes in pro-inflammatory cytokine levels (TNF-α, IL-1β and IL-6), but these effects were not associated with any changes in clinical symptoms [237].

#### 4.2.2. Melatonin and Multiple Sclerosis

Multiple sclerosis (MS), a progressive neurodegenerative disorder triggered by an autoimmune response against myelin [238], is the most common neurological disease in young adults, with a worldwide prevalence of 1.1–2.5 million cases [239]. Although the etiology of MS is not currently fully understood, one environmental factor that appears to be implicated is latitude, as the prevalence of the disease increases in northern countries [240]. This might be associated with a reduction in daylight exposure [241,242], as a diminished prevalence of MS has been described in mountainous areas with respect to neighboring lower areas [243]. Recently, shiftwork at a young age has been associated with increased incidence of MS, with a positive correlation between the risk of MS and the duration of shift work [244].

MS patients exhibit impaired circadian rhythms for both melatonin and aMT6. A high percentage of patients with exacerbated MS were shown to display an inverted melatonin circadian rhythm [245] and alterations in aMT6 urine excretion, specifically lower total urine aMT6 levels than healthy controls. Additionally, patients exhibited significantly reduced night-time excretion of aMT6 when compared with controls, normalized by IFN-β treatment [246]. In addition to studies of melatonin action over the course of the disease, endogenous melatonin has been related to the clinical complications of MS. Serum melatonin levels inversely correlate with depression in MS patients [247]. Additionally, diurnal vision impairment related to MS was shown to be linked to the melatonin circadian rhythm; more importantly, it was improved by oral treatment with melatonin [248].

Although some epidemiological studies might indirectly suggest a detrimental effect for melatonin in MS, studies using an experimental autoimmune encephalomyelitis (EAE) model have reported contradictory results. Luzindole administration suppressed EAE [249] and pinealectomy showed an age-dependent effect on EAE; when new-born rats were pinealectomyzed, they suffered extensive pathological damage and severe neurological deficits after EAE induction, but adult pinealectomyzed rats were protected against the development of EAE [250]. Moreover, exogenous melatonin reduced both the severity and the duration of EAE-induced paralysis in rats and diminished both macrophage and CD4+ and CD8+ T cell infiltration in the spinal cord and ICAM-1 expression in the blood vessels close to EAE lesions [251].

#### 4.2.3. Melatonin and Systemic Lupus Erythematosus

Systemic lupus erythematosus (SLE) is a multifactorial autoimmune disorder with a yearly incidence of 1 to 10 and an estimated prevalence of approximately 20–150 cases per 100,000 people. A hallmark of SLE is the formation of immune complexes in blood and tissues that cause extensive tissular damage. The sustained immune response causes lupus patients to develop localized inflammatory episodes that give rise to a vicious circle in which the autoimmune response leads to a number of immunological abnormalities and tissue destruction [252].

An uncoupled melatonin circadian rhythm has been described in lupus-prone mice [253]. However, melatonin levels and seasonal light variations show no association with disease activity in SLE patients from a subarctic region [254]. Melatonin administration exerts contradictory effects in experimental models of SLE; it has been shown to be beneficial or deleterious depending on parameters such as timing of the administration and gender. Melatonin administered in the morning increased the survival of lupus-prone animals, although the effect was not reproduced after evening treatment [255]. Additionally, melatonin reduced vascular lesions and inflammatory infiltration in the kidneys, diminished titers of anti-collagen II and anti-dsDNA autoantibodies, reduced the production of pro-inflammatory cytokines and increased the release of anti-inflammatory cytokines in both female lupus-prone animals [256] and pristane-induced lupus [257]. However, melatonin had no effect or actually worsened the disease in male lupus-prone mice [256].

#### 4.2.4. Melatonin and Type 1 Diabetes

Type 1 diabetes (T1D) is an autoimmune disease in which the immune response against pancreatic β-cells causes an insulin deficit. Although T1D only accounts for 5%–10% of all cases of diabetes, its incidence is rising worldwide [258]. As for most autoimmune pathologies, a latitudinal gradient of T1D incidence has been demonstrated, with cases increasing with latitude. It has been proposed that this relationship is associated with UV exposure and vitamin D levels [259], although melatonin involvement cannot be ruled out. Interestingly, low levels of insulin in experimental models of T1D correlate with increased production of melatonin, which is normalized by insulin administration [260]. Pinealectomy of newborn non-obese diabetic (NOD) mice, a mouse model of autoimmune T1D, was shown to significantly diminish survival and induce glycosuria, whereas chronic administration of melatonin increased survival and delayed the onset of the disease, facilitating the maintenance of normal plasmatic glucose levels [261]. Moreover, melatonin reduced the proliferation of splenocytes and Th1 cells, prolonging the survival of pancreatic islet grafts in NOD mice [262]. Melatonin is not only useful for preventing the development of T1D but also shows protective effects against diabetes-associated cardiovascular disturbances by improving vascular contractile performance in diabetic rats [263] and reducing blood pressure in T1D teenagers [264].

#### 4.2.5. Melatonin and Irritable Bowel Syndrome/Inflammatory Bowel Disease

Inflammatory bowel disease (IBD) encompasses a group of inflammatory diseases that includes ulcerative colitis and Crohn’s disease; for these two diseases, both the innate and adaptive immune responses appear to be implicated [265]. Irritable bowel syndrome (IBS) is a functional gastrointestinal disorder associated with visceral hypersensitivity and abnormal gastrointestinal motor functions [266]. A relationship between impaired melatonin synthesis and secretion and IBS has been suggested, as urinary levels of aMT6 are lower in IBS patients than in healthy controls. In support of this, altered melatonin levels have been shown to be important for the disease, and some clinical trials have demonstrated the beneficial effects of the agent in reducing abdominal pain [267,268] and distension [267] and improving rectal sensibility [268], IBS scores and quality of life [269,270].

The impairment of circadian rhythmicity has also been shown to be related to the course of IBD in experimental models. Mice subjected to continuous changes of the light/dark cycle or to sleep deprivation have been shown to exhibit more severe colitis with increased weight loss and mortality compared to control animals [271,272]. Furthermore, melatonin promoted beneficial effects in several experimental models of colitis in rodents. It reduced visceral hyperalgia [273] and diminished disease severity [274,275] by antioxidant mechanisms, reducing lipid peroxidation and nitrosative stress [275,276] and protecting endogenous antioxidants from depletion [118]. Moreover, melatonin was shown to modulate the immune attack on the colonic mucosa by regulating the activities of macrophages [277] and matrix metalloproteinases (MMP) 2 and 9 [127] and by suppressing iNOS and COX-2 activities [276], pro-inflammatory cytokine levels [118,128] and adhesion molecules [124]. Furthermore, melatonin has been shown to modulate apoptosis in colitis models [128]. Intracellular actions such as NF-κB inhibition and c-Jun activation have also been associated with the effects of melatonin on colitis [124,128].

Although available data regarding melatonin treatment in experimental models of colitis suggest positive effects, to date, no clinical trials have been conducted to investigate the effects of melatonin on IBD. Only three case reports have been published, each with different results. While a patient suffering ulcerative colitis observed that melatonin caused the disappearance of clinical symptoms and that these symptoms reappeared when melatonin consumption stopped [278], the other two cases, one an ulcerative colitis patient and the other a patient with Crohn’s disease, experienced an exacerbation of their respective diseases, which remitted after melatonin intake ceased [279,280].

### 4.3. Melatonin and Vaccination: A Worthwhile Area to Explore

Vaccines have been successfully used to establish or improve immunity to a particular disease. The immunity-promoting activity of a vaccine is not only determined by the particular antigenic component utilized but also by the addition of suitable adjuvants that are capable of activating and promoting an efficient immune response against the infectious agent of interest. Based on the immunoregulatory properties of melatonin, some *in vivo* studies have explored its use in vaccination (Scheme 1). Melatonin was shown to increase the humoral responses of sheep vaccinated against *Dichelobacter nodosus*[281], which is the bacteria that causes ovine footrot, a major cause of lameness in sheep [282]. In this study, the administration of subcutaneous slow-release melatonin implants (18 mg/animal given 21 days after the first dose of vaccine) increased antibody titers in synergy with aluminum hydroxide. Double doses of melatonin notably increased antibody titers and IgG levels compared to untreated, vaccinated animals [283]. The administration of melatonin either via implants or injections also enhanced the platelet response to thrombin stimulation, improving the percentage and rate of aggregation and lag-time [284]. The beneficial effects of melatonin on the immune response to vaccination against *Clostridium perfringens type D* in sheep have also been described [285]. Interestingly, the highest increases in serum antibody levels due to melatonin were observed when vaccination took place prepartum, suggesting that the time of immunization plays an important role in determining the effects of melatonin on the immune response. The powerful immune response originating after the administration of melatonin via implants might be explained through two primary mechanisms: melatonin could effectively augment the antibody response by enhancing antigen presentation to immunocompetent cells [18,89], or melatonin could modulate cytokine production at the beginning of the immune response and could therefore control important cellular responses. The potential role of melatonin as an adjuvant has also been suggested in studies examining vaccines developed against prostate cancer [286].

The goal of an immunization program should not only be restricted to protection against infection but should also include modulation of the pathology inflicted by the agent. To evaluate this, the administration of melatonin in combination with different immunization regimens using various *Schistosoma mansoni* antigens in hamsters (with schistosomiasis) was evaluated [287]. Melatonin provided excellent enhancement of vaccine action with cercarial and soluble worm antigens; this was accompanied by significant improvements in GSH levels. Taking into account its antioxidant capacity [288], melatonin protected against inflammation associated with Aβ vaccination via direct and indirect actions [289], suggesting that it could also be an effective adjuvant in vaccines developed for immunotherapy of Alzheimer’s disease (AD). Immune cell functions are strongly influenced by the antioxidant/oxidant balance; thus, antioxidant levels in these cells play a pivotal role in protecting against oxidative stress and preserving adequate cellular function. The immunoregulatory and antioxidant properties of melatonin were also demonstrated in an open-field vaccination procedure in sheep against *D. nodosus*[290]. In this study, the co-administration of melatonin with footrot vaccine neutralized the rise of serum NO found in vaccinated animals. This effect could be explained by the direct action of melatonin on NO or by its known inhibitory actions against iNOS activity.

### 4.4. Immunological Aspects of Melatonin in Transplantation

All experimental studies performed in models of perfused organ transplantation have shown the beneficial effects of melatonin in prolonging graft survival (Scheme 1). Indeed, treatment with high doses of melatonin (200 mg/kg/bw) were shown to inhibit the immune response to allografts by reducing the proliferation capacity of lymphocytes, preventing rejection and doubling allograft survival in a rat cardiac transplant model [291]. Similar protective effects were observed in rat lungs following reperfusion injury after prolonged ischemia [292]. High doses of melatonin reduced the proportion of Th1 cells and elevated the percentage of IL-10-producing cells, significantly prolonging pancreatic islet graft survival in NOD mice [262]. The immunosuppressive effects of melatonin supplementation were also evident in a model of autologous intraperitoneal ovary transplantation in rats. A single application of melatonin attenuated ovarian tissue necrosis following engraftment [293]. In line with this finding, a more recent study reported a reduction of apoptosis in human ovarian grafts in melatonin-treated hosts [294]. The reductions in both apoptosis [294] and necrosis [293] might be related to the anti-apoptotic and antioxidant properties of melatonin. Another potential mechanism by which melatonin can exert beneficial effects following transplantation is the inhibition of cellular damage caused by surgical stress and ischemia-reperfusion injury (IRI). This has been demonstrated in animal models of hepatic IRI in which melatonin supplementation exerted a protective effect in the liver [295]. Specifically, melatonin reduced neutrophil recruitment, increased GSH and decreased oxidative stress. Furthermore, the number of apoptotic cells was reduced after melatonin administration [296]. Therefore, melatonin might reduce graft immunogenicity following transplantation, directly improving clinical outcome.

The usefulness of melatonin as an additive for increasing the quality of organ preservation solution has also described. In a recent study, the addition of melatonin to Institute Georges Lopez (IGL-1) solution improved non-steatotic and steatotic liver graft preservation, limiting their risk for cold IRI [297]. The benefits of melatonin correlated with the generation of NO and the prevention of oxidative stress and inflammatory cytokine release, including TNF-α and adiponectin. Recently, melatonin was used in multidrug donor preconditioning (MDDP), which improved liver preservation and completely prevented hepatic reperfusion injury [298,299]. This MDDP protection was provided by the antioxidative, anti-inflammatory and anti-apoptotic actions of melatonin. The immunosuppressive potential of melatonin is also evident, as immunosuppressive maintenance therapy with cyclosporine A was shown to increase midnight melatonin levels [300].

### 4.5. Melatonin and Immunosenescence

Aging reflects the sum of the progressive changes that occur in several key physiological systems, including the immune system, which is continuously remodeled over the course of life, a process known as immunosenescence. Many age-related pathophysiological conditions, such as increased susceptibility to infectious diseases, neoplasias, metabolic diseases, osteoporosis and autoimmune diseases, are directly associated with immunosenescence [301]. It is interesting to note that many hormones that are associated with the maintenance of immune function also decline with advancing age and that the relationship between the endocrine system and the immune system is considered to be of crucial importance in normal human physiology and in mediating age-associated degenerative diseases [302,303]. The decline in the production of a number of hormones associated with aging, such as melatonin, has been proposed to play a significant role in contributing to immunosenescence [302]. With some exceptions [304,305], the age-associated decline of melatonin has been repeatedly reported [306–311] and usually overlaps with age-related impairment of the immune system.

Although there are many studies that have demonstrated the immunomodulatory properties of melatonin, there have been only a limited number of investigations related to immunosenescence (Scheme 1). The administration of melatonin in normal or immunocompromised mice was shown to result in elevated antibody responses *in vitro* and *in vivo*[312]. Significantly, loss of thymocytes with age is the main cause of structural thymic atrophy and thymic weight loss. However, melatonin administration rejuvenated degenerated thymuses and corrected peripheral immune dysfunctions in aged mice [79]. This reversal of age-related thymic involution by melatonin could be attributable to increased thymic cellularity due to its anti-apoptotic and proliferation-enhancing effects [313]. The antioxidant activity of melatonin and its metabolites may also account for its anti-apoptotic effects on immune cells [314,315]. In fact, melatonin is able to delay endoplasmic reticulum stress-induced apoptosis in aged leukocytes and may counteract age-related degenerative phenomena linked to oxidative stress at the cellular level [316]. It was shown that age-related increases in oxidative burden were reversed by melatonin in golden hamsters, improving general immune status [317]. In another study, injections of melatonin restored immune functions in experimentally immunosuppressed or aging mice. Here, melatonin was able to enhance the antibody response to a T-dependent antigen. Moreover, the enhanced antibody response was associated with an increased induction of T helper cell activity and IL-2 production [318].

Additionally, melatonin has been demonstrated to exert inhibitory effects on various parameters of immune function. Melatonin has been shown to inhibit the production of pro-inflammatory cytokines, suggesting that indoleamine may help to reduce acute and chronic inflammation. Indeed, melatonin was able to reduce inflammation in the livers of senescence-accelerated prone (SAMP8) mice by decreasing the mRNA and protein expression levels of TNF-α and IL-1β and increasing the IL-10 expression level [319]. Similar data were also obtained with 24-month-old rats in which melatonin significantly reduced the levels of the pro-inflammatory cytokines TNF-α, IL-1β and IL-6 in the liver [122]. The immunomodulatory effects of melatonin in aging are also evident in the central nervous system (CNS), as dietary melatonin was shown to selectively reverse the lack of response to an inflammatory stimulus in the brains of aged mice [320].

Based on the experimental data that have been accumulated and considering its lack of toxicity [321], high lipophilicity and great capacity to prevent cell damage [167], melatonin is one of the most attractive agents that has been investigated in relation to age-associated deterioration of the immune system and should be considered as a potential agent to improve quality of life in a rapidly aging population.

## 5. Conclusions

Currently, the role of melatonin as an effector that can modulate the immune system is undeniable. The almost ubiquitous distribution of melatonin receptors and its synthesis by the immune system underpin the melatonin/immune system relationship. The effects of several immunological mediators on melatonin production close the bidirectional circuit.

**Scheme 2:**
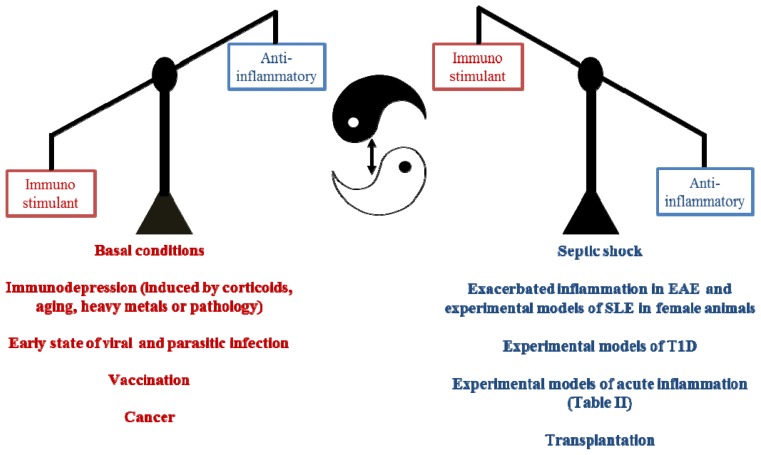
Comprehensive graphic scheme depicting how melatonin buffers the immune system.

In this review, we have highlighted the fact that melatonin acts on both the innate and specific responses of the immune system via combined mechanisms that mainly involve the modulation of cytokines and the production of oxidative stress. Overall, melatonin might act as an immunostimulant under basal or immunosuppressed conditions, providing a pre-activated state for a more effective early immune response against external stressors, such as viruses and parasites. However, in the presence of a transient or chronic exacerbated immune response, such as septic shock, melatonin might exert negative regulation and could be considered an anti-inflammatory molecule. Therefore, we have coined the termed “immunological buffer” in an attempt to define the pleiotropic, varied and complex effects of melatonin on the immune system in the most accurate way (Scheme 2).

The various immunomodulatory actions exerted by melatonin have been successfully elucidated in several *in vivo* experimental models of inflammation, infection, sepsis or immunosenescence, and there has been general agreement that melatonin can be applied with negligible side effects. These conclusions, along with the future implementation of working models, such as melatonin knockout animals or highly specific monoclonal antibodies, will allow us to properly determine the scope of the pathophysiological role of melatonin to initiate more ambitious clinical trials. However, this will only be successful with firm commitment from both clinical researchers and companies to facilitate the progress of melatonin research from the bench to the patient bed.

## Figures and Tables

**Table 1:** Distribution and role of melatonin receptors in the immune system. BALT: bronchus-associated lymphoid tissue; IL: interleukin; IP3: inositol triphosphate; PBMCs: peripheral blood mononuclear cells. Empty spaces indicate unknown function.

	Receptor	Distribution	Effector mechanism	References
**Membrane receptors**	MT_1_	Human PBMCs	Regulation of IL-2 production Regulation of cAMP levels	[46–48]
		Different subsets of human T lymphocytes, B cells and monocytes		[46]
		Jurkat (human T cells)	Regulation of IL-2 production and IL-2R (CD25) levels Regulation of cAMP levels	[44,48–52]
		U937 (human monocyte cells)		[50]
		Mouse thymus		[53]
		Mouse spleen		[53]
		Mouse peritoneal macrophages	Regulation of cAMP levels	[51]
		Rat thymus		[54]
		Rat spleen		[54]
		Rat B cells		[54]
		Rat CD4^+^, CD8^+^ and CD4^+^ CD8^+^ thymocytes		[54]
		RBL-2H3 (rat mast cells)		[45]
		Spleen of palm squirrel	Regulation of organ weight Regulation of splenocyte proliferation	[55–59]
		Thymus of palm squirrel	Regulation of thymocyte proliferation Regulation of IL-2 production	[55,57,59]
		PBMCs of palm squirrel		[59]
		BALT of quail		
	MT_2_	Jurkat		[44]
		Mouse thymus		[53]
		Mouse splenocytes	Increased proliferation	[60]
		Rat thymus		[61]
		Rat spleen		[61]
		Rat leukocytes	Inhibition of leukocyte rolling	[62]
		RBL-2H3		[45]
		Chicken spleen	Regulation of splenocyte proliferation Regulation of cAMP and IP3 levels	[41]
		Chicken thymus		[63]
		Chicken lymphocytes		[63]
		BALT of quail		[64]
		Spleen of quail		[65]
	MT_1_/MT_2_	Human PBMCs	Inhibition of TNFα-induced apoptosis Activation of ERK signaling pathway	[66]
**Nuclear receptors**	RZRα	Human PBMCs, including different subsets of T lymphocytes, B cells and monocytes		[46,67]
		Jurkat		[49]
		RPMI 1788 and P16 (human B cells)	Repression of 5-LOX expression	[68]
		HL-60 (promyelocytes)		[68]
		Mono Mac 6 (monocytes)		[68]
	RORα	Human PBMCs		[48]
		Human cytotoxic T lymphocytes [RORα1]		[46]
		Human PBMCs, including different subsets of T lymphocytes, B cells and monocytes [RORα2]		[46]
		Jurkat [RORα1, RORα2, RORα3]		[49,50,69]
		U937 [RORα1, RORα2]	Regulation of IL-6 production	[50]
		RPMI 1788 and P16 [RORα2, RORα3]		[68]
		HL-60 [RORα2, RORα3]		[68]
		Mono Mac 6 [RORα2, RORα3]		[68]
		Mouse thymus and spleen		[53]
	RZR/ROR	Human PBMCs	Regulation of IL-2 and IL-6 production and of IL-2R (CD25)	[51,70,71]
		Jurkat	Regulation of IL-2 production	[44,49]
		U937	Regulation of IL-6 production	[72]

**Table 2:** *In vivo* effects of melatonin under basal and immunosuppressed conditions.

Immune condition	Melatonin effects	Melatonin administration	References
Basal	Increases lymphocyte counts and the blastogenic stimulation ratio of spleen and thymus Increases cellularity in thymus and spleen	25 mg/kg to adult male squirrels for 60 consecutive days during May–June	[77]
Basal	Splenic hypertrophy and extramedullary hematopoiesis	25 mg/kg to adult male Syrian hamsters kept under a long photoperiod	[78]
Basal	Increase in cell numbers of macrophages/microglia and upregulation of MHC and CD4 antigens	Multiple daily injections of melatonin in the pineal gland and different regions of 1-day-old rat brains for 7–11 days	[84]
Basal	Increases bone marrow NK cells and monocytes	Daily administration through diet (7–14 days) to young adult male mice	[85]
Basal	Increases neutrophil chemotactic response to a physiological chemoattractant and the expression of intracellular chemokines	20 mg daily to human volunteers	[88]
Basal	Increases in splenocyte proliferation and IL-2 and IL-1β levels	500 mg/kg to young mice	[91]
Aging-induced immunosuppression	Reverses thymic and splenic involution, total numbers of thymocytes and splenocytes, mitogen responsiveness and NK cell activity	15 mg/kg in drinking water to 22-month-old female C57BL mice for 60 consecutive days	[79]
Aging-induced immunosuppression	Increases humoral response (IgG1 and IgM levels)	Subcutaneous injection of 10 mg/kg for 7 days to 28-month-old male Wistar rats	[106]
Aging-induced immunosuppression	Increases total leukocyte and lymphocyte counts, mitogenic response of splenocytes and delayed type hypersensitivity response	Daily subcutaneous administration of 0.25 mg/kg to squirrels	[107]
Aging-induced immunosuppression	Increases B cell proliferation and Th1 cytokines and decreases Th2 response	Injection of old (16.5 months) female C57BL/6 mice	[108]
Immunosenescence induced by aging plus ovariectomy	Restores impaired chemotaxis, mitogenic response, IL-2 release and NK cell activity	1 mg/kg in drinking water to Wistar albino female rats	[87]
Dexamethasone-induced immunosuppression	Restores decreased thymus and spleen activities, lymphoid tissues mass, total leukocyte counts and bone marrow and T cell-mediated immune function	25 mg/kg to adult squirrels along with dexamethasone for 60 consecutive days	[80]
Dexamethasone-induced immunosuppression	Enhances IL-2 production and thymic and splenic lymphocyte proliferation by membrane receptor-mediated mechanism	Daily subcutaneous administration of 0.25 mg/kg to squirrels during evening hours	[59]
Corticosterone-induced immunosuppression	Antagonizes the depression of antibody production	Evening injections to mice	[109]
Immunosuppression due to propranolol- and PCPA-induced pineal inactivation	Reverses the suppression of the humoral response and autologous mixed lymphocyte reaction	Evening injections to mice	[109]
Immunosuppression by trauma-hemorrhage	Improves reduced IL-1, IL-2 and IL-6 release and splenocyte proliferative capacity	Subcutaneous injection of 10 mg/kg in the evening of the day of surgery (soft-tissue trauma and hemorrhagic shock) and on the following evening in C_3_H/HeN mice	[92]
Lead-induced immunotoxicity	Increases thymus weight and splenic T and B cell and NK, T and B cell functions	10 or 50 mg/kg orally administered to ICR mice daily for 28 days, 2 h before Pb treatment	[110]

PCPA: p-chlorophenylalanine; Pb: lead; Ref: reference.

**Table 3:** *In vivo* effects of melatonin on inflammatory conditions.

	Inflammatory condition	Melatonin effects	Molecular mechanism	References
**Rat**	Exercise-induced cardiac inflammatory injury	Reduces TNF-α, IL-1β and IL-6 production		[111]
	Experimental colitis	Counteracts the high production of TNF-α, IL-1 and NO	Downregulation of NF-κB	[112]
	Diabetes-associated low-grade inflammation	Lowers TNF-α, IL-6 and CRP		[113]
	Experimental model of traumatic brain injury	Reduces upregulation of IL-6, iNOS, SOCS-3 and oxidative stress	Overcomes STAT-1 inactivation	[114]
	Heatstroke-induced multiple organ dysfunction syndrome	Attenuates high production of TNF-α, IL-1β and IL-6 and promotes IL-10 production		[94]
	Ischemia reperfusion-induced liver damage	Attenuates enhanced levels of TNF-α, IL-6 and NO	Suppresses the increase in MyD88, ERK, phosphorylated JNK and c-Jun and nuclear translocation of NF-κB	[115]
	Neuroinflammation in experimental diabetic neuropathy	Attenuates elevated levels of TNF-α, IL-6, iNOS, COX-2 and oxidative stress	Decreases NF-κB cascade activation	[116]
	FK506-induced nephropathy	Lowers TNF-α, IL-6 and NO levels		[117]
	Acetic acid-induced colitis	Reverses increased levels of TNF-α, IL-1β, IL-6, MPO and oxidative stress		[118]
	Cerulein-induced pancreatitis	Reduces expression of TNF-α, IL-1β, IL-6, IL-8 and iNOS	Inhibition of elevated nuclear binding of NF-κB	[119]
	Hemorrhagic shock	Suppresses the release of TNF-α and IL-6		[120]
	DMN-induced liver injury	Decreases expression of TNF-α, IL-1β, IL-6 and iNOS	Inhibition of increased nuclear binding of NF-κB	[121]
	Aging	Attenuates increased levels of TNF-α, IL-1β, IL-6, iNOS and LPO		[122]
	Cecal dissection-induced bacterial peritonitis	Lowers levels of TNF-α, IL-6 and MDA		[123]
	Pancreatic fluid-induced lung inflammation and airway hyperreactivity	Reduces TNF-α and iNOS concentrations		[95]
	TNBS-induced colitis	Decreases high levels of TNF-α, IL-1 and NO	NF-κB inhibition and blockade of IκBα degradation	[112,124]
	Neuro-inflammation induced by intra-cerebroventricular administration of LPS	Decreases TNF-α, IL-1β and oxidative stress		[125]
	Experimental periodontitis	Reduces TNF-α, IL-1β and MDA		[126]
	DNBS-induced colon injury	Reduces expression of TNF-α and MMP-9 and MMP-2 activities	Reduction in NF-κB activation and phosphorylation of c-Jun	[127,128]
	Taurocholate-induced acute pancreatitis	Reduces TNF-α and amylase levels		[129]
	*Escherichia coli*-induced pyelonephritis	Reverses increased levels of TNF-α and MDA		[130]
	Spinal cord injury	Decreases expression of TNF-α and MMP-9 and MMP-2		[127]
	Lung ischemia-reperfusion injury	Diminishes levels of TNF-α	Inhibition of NF-κB protein levels	[131]
	Hypoxia-induced retinal ganglion cell death	Reverses the upregulation of TNF-α, IL-1β and LPO		[132]
	Mechlorethamine-induced nephrotoxicity	Ameliorates the increased production of TNF-α and IL-1β		[133]
**Mouse**	Immunological liver injury	Attenuates the increases in TNF-α and IL-1β		[134]
	Radiation-induced lung injury	Reduces TNF-α, TGF-1 and oxidative stress		[135]
	Maternal LPS-induced inflammation	Attenuates elevation of TNF-α in maternal serum and fetal brain		[136]
	Indomethacin-induced chronic gastric ulcer	Blocks expression of TNF-α, IL-1β and IL-8	Inhibition of ERK and JNK phosphorylation and NF-κB, c-Fos and c-Jun expression	[137]
	Alzheimer’s transgenic mice	Decreases TNF-α levels in hippocampus		[138]
**Human**	Infant endotracheal intubation	Lowers IL-6, IL-8, IL-10 and IL-12		[139]
	Strenuous exercise	Prevents overexpression of TNF-α, IL-6, IL-1ra and oxidative stress		[140]
	Duchenne muscular dystrophy	Reduces levels of TNF-α, IL-1β, IL-6, IL-2, IFN-γ and oxidative stress		[141]
	Respiratory distress syndrome	Limits serum rise in TNF-α, IL-6 and IL-8		[142]

CRP: C-reactive protein; MPO: myeloperoxidase; NO: nitric oxide; DMN: dimethylnitrosamine; TNBS: 2,4,6-trinitrobenzenesulfonic acid; DNBS: dinitrobenzene sulfonic acid; empty spaces indicate unknown molecular mechanisms.
